# Identification of a Cytotoxic Form of Dimeric Interleukin-2 in Murine Tissues

**DOI:** 10.1371/journal.pone.0102191

**Published:** 2014-07-14

**Authors:** Lucile E. Wrenshall, Suzanne E. Clabaugh, David R. Cool, Prakash Arumugam, William C. Grunwald, Deandra R. Smith, Gino C. Liu, John D. Miller

**Affiliations:** 1 Department of Neuroscience, Cell Biology, and Physiology, Boonshoft School of Medicine Wright State University, Dayton, Ohio, United States of America; 2 Department of Pharmacology and Toxicology, Boonshoft School of Medicine, Wright State University, Dayton, Ohio, United States of America; 3 Eppley Institute for Cancer Research, University of Nebraska at Omaha, Omaha, Nebraska, United States of America; Center for Cancer Research, National Cancer Institute, United States of America

## Abstract

Interleukin-2 (IL-2) is a multi-faceted cytokine, known for promoting proliferation, survival, and cell death depending on the cell type and state. For example, IL-2 facilitates cell death only in activated T cells when antigen and IL-2 are abundant. The availability of IL-2 clearly impacts this process. Our laboratory recently demonstrated that IL-2 is retained in blood vessels by heparan sulfate, and that biologically active IL-2 is released from vessel tissue by heparanase. We now demonstrate that heparanase digestion also releases a dimeric form of IL-2 that is highly cytotoxic to cells expressing the IL-2 receptor. These cells include “traditional” IL-2 receptor-bearing cells such as lymphocytes, as well as those less well known for IL-2 receptor expression, such as epithelial and smooth muscle cells. The morphologic changes and rapid cell death induced by dimeric IL-2 imply that cell death is mediated by disruption of membrane permeability and subsequent necrosis. These findings suggest that IL-2 has a direct and unexpectedly broad influence on cellular homeostatic mechanisms in both immune and non-immune systems.

## Introduction

Interleukin-2 (IL-2) is a fascinating cytokine, with widely varying functions including promotion of apoptosis, proliferation and survival of lymphocytes [1]. Not surprisingly, these varied responses depend on the type of lymphocyte, and on the activation state of the cell. Apoptosis, for example, occurs in activated T cells that are exposed to IL-2 and then re-activated [2]. This activation-induced cell death (recently renamed restimulation-induced cell death) is thought to be a feedback mechanism designed to limit the expansion and facilitate the down-regulation of antigen-specific immune responses [3]. The importance of restimulation-induced cell death is demonstrated by IL-2 knock out mice, which develop a lethal lympho-proliferative phenotype and autoimmunity [4].

While IL-2 is typically considered a monomeric protein, studies by Eitan, et al described a dimeric form of IL-2 that was cytotoxic to oligodendrocytes [5]. This dimeric IL-2, extracted from fish optic neurons, was thought to be the result of the cross-linking of two IL-2 monomers by optic nerve-derived transglutaminase. This hypothesis was based on data demonstrating that recombinant human IL-2 also formed a cytotoxic dimer after exposure to the same transglutaminase [6]. Dimeric IL-2 was shown to induce apoptosis of oligodendrocytes after several hours, likely through a p53-related mechanism [7].

Our laboratory recently reported that IL-2 is retained in the blood vessel wall by heparan sulfate [8], Specifically, we showed that heparanase digestion of murine aortic tissue resulted in the release of biologically active, monomeric (15 kD) IL-2 [8]. Interestingly, heparanase digestion also resulted in the release of a 30 kD (dimeric) form of IL-2. The dimeric form of IL-2 was isolated from murine aortas and found to be cytotoxic to several different cell types expressing the IL-2 receptor. In contrast to the studies by Eitan, et al, the onset of cell death was rapid and dimer-treated cells appeared to be dying by oncosis, which is characterized by a loss of membrane integrity and cellular swelling [10]. These results demonstrate that dimeric IL-2 is present endogenously in mammalian tissues and suggests that so positioned, dimeric IL-2 may function to restrict excess proliferation under pro-inflammatory conditions in vivo.

## Materials and Methods

### Materials and cell lines

Murine aortas were obtained from Balb/c mice. Small sections of human iliac artery were obtained from deceased donor organs. Heparinase I and chemical reagents, unless otherwise indicated, were obtained from Sigma-Aldrich (St. Louis, MO). Alpha-Cyano-4-Hydroxy-Cinnamic Acid (CHCA) MALDI Matrix was from Thermo Scientific (Waltham, MA). Recombinant mouse IL-2 was from Cell Sciences (Canton, MA). The fluorescent dye used to label IL-2 (800CW) was obtained from LI-COR Biosciences (Lincoln, NE). CellTox Green cytotoxicity and CellTiterGlo proliferation assays were from Promega (Madison, WI). LDH cytotoxicity assay was from Roche (Indianapolis, IN). The following antibodies were used: rabbit anti-mouse/human IL-2 receptor (IL-2R) βpolyclonal antibody (Novus Biologicals, Littleton, CO), mouse anti-rat IL-2Rβ monoclonal antibody (clone L316, AbD Serotec, Raleigh, NC), rat anti-mouse blocking monoclonal antibody (clone S4B6, BD Biosciences, San Jose, CA), and a chicken polyclonal antibody recognizing human and murine IL-2 (Sigma). CTLL-2 (mouse cytotoxic T lymphocyte), NRK (normal rat kidney epithelium), HK-2 (human kidney epithelium), B16-F10 (mouse melanoma) cells, and EL4.IL-2 (murine lymphoma) cell lines were obtained from American Tissue Type Collection (Manassas, VA). Smooth Muscle Cell Medium was from ScienCell (Carlsbad, CA).

### Ethics statement

Mice were housed and treated in strict accordance with protocols approved by the Wright State University Laboratory Animal Care and Use Committee (AUP # 891). Authorization for use of donated human tissue for research was obtained from the donor (primary authorization) or next of kin. Tissues were provided by Life Connection of Ohio (www.lifeconnectionofohio.org). Human subjects are defined as living individuals and therefore use of these tissues is exempt from Institutional Review Board review at Wright State University. Human vascular smooth muscle cells were grown in accordance with protocols approved by the Institutional Biosafety Committee (IBC# 230).

### Dye-labeled IL-2

Murine IL-2 (R & D Systems) was covalently conjugated to an activated infrared dye (800CW, LI-COR) per the manufacturer's instructions. Briefly, activated dye was added to the IL-2 at a molar ratio of 1∶1. Following a 2 hour incubation at 24°C, unconjugated dye was removed from the preparation using a de-salting column. The concentration of the dye-conjugated IL-2 was then determined by Bradford assay (Sigma). To confirm conjugation of the IL-2 to the 800CW dye, 0.5 µg of dye-IL-2 was separated by SDS-PAGE, transferred to nitrocellulose, and analyzed on an Odyssey infrared scanner (LI-COR Biosciences).

### Cytotoxicity assays

Human vascular smooth muscle cells (VSMC) were incubated with dimeric IL-2 at the times and concentrations indicated in the figure legends. Cell death was assessed by either LDH release or by fluorescence of a DNA-binding dye (CellTox Green). For assessment of LDH release, cell culture media (80 µL/well) was harvested and centrifuged at 4,000 rpm for 10 minutes to remove debris. LDH activity in the supernatants was quantified using a commercially available kit (Roche). CellTox Green, designed to assess cell death kinetically, was added just prior to or simultaneously with dimeric IL-2 or vehicle control. Absorbance or fluorescence was read at the appropriate wavelength using a Synergy H1 microplate reader (Biotek, Winooski, VT). In select experiments, cells were pre-incubated for 15 minutes with blocking anti-IL-2Rβ (rabbit anti-human polyclonal, Novus) or anti-IL-2 antibodies (S4B6, BD Biosciences).

### CTLL-2 assay

CTLL-2 cells were cultured in RPMI-1640 media with 10% FBS. For bioassay, cells were plated in 96 well plates at 5×10^4^ cells/ml, and cultured with increasing concentrations of IL-2 for 24 h at 37°C. Proliferation was assessed by ATP content per manufacturer's instructions (CellTiterGlo).

### Isolation of dimeric IL-2 from tissues or media

Murine aortas were cleaned of adventitia and adipose, cut into ∼1 mm^3^ pieces, and homogenized in ice-cold in urea extraction buffer (8 M urea, 100 mM Tris-HCl, pH 7.5). The extracts were sonicated on ice and then clarified by centrifugation. The resultant supernatants were separated by reducing SDS-PAGE (3–15% gradient; Bio-Rad) and then subjected to negative zinc staining (Bio-Rad). Using a companion Western blot of the extract probed with a chicken polyclonal anti-IL-2 antibody (Sigma) as a guide, the 30 kD bands were excised and electro-eluted (Bio-Rad). Eluted samples were pooled, dialysed over a period of 48 hours with four buffer exchanges, concentrated by dialysis against aquacide II (Calbiochem) or ammonium sulfate precipitation, then re-suspended in storage buffer (TBS without Ca or Mg) and stored at 4C. The amount of dimeric IL-2 isolated was quantified by Western blot analysis with a chicken polyclonal anti-IL-2 antibody (Sigma) incorporating scanning densitometry and a standard curve of known concentrations of monomeric IL-2.

EL4.IL-2 cells were stimulated for 48 h with 1 µg/ml PHA and 10 ng/ml PMA. The supernatant was collected, passed through an Amicon filter (0.2 µM), then subjected to reducing SDS-PAGE and electro-elution as described above. Isolation of 30 kD IL-2 was verified by Western blot analysis and quantified by scanning densitometry.

### Vascular smooth muscle cell cultures

Human vascular smooth muscle cells (VSMC) were isolated from pieces of aorta using a modification of a previously reported technique [11]. Briefly, approximately 1 cm pieces of aorta were washed with PBS and all adipose was removed. The artery was sliced to open the lumen, and small (1 mm×2 mm) pieces were cut and placed lumen side down into a 75 cm tissue culture flask (6–8 pieces per flask). The tissue (without media) was incubated at 37°C for 3–4 h to allow the tissue to dry and adhere to the flask. Ten mls of SMC media (ScienCell) were carefully added to the flask so as not to dislodge the pieces of aorta. The tissue was maintained at 37°C and SMCs were harvested in 3–4 weeks. VSMC were then passaged every 7 days and used after 4 passages [8].

### Western blot analysis

Western blot analysis of supernatants was performed as follows. EL4.IL2 supernatants were generated as described above. An equal volume of 2X Laemmli running buffer (Bio-Rad, Hercules, CA) containing 5% v/v β-mercaptoethanol (Bio-Rad) was added to the media which was then separated by sodium dodecyl sulfate polyacrylamide gel electrophoresis (SDS-PAGE) on 10% gels (Bio-Rad), transferred to 0.2 µm nitrocellulose (Bio-Rad), blocked with Tris-buffered saline (TBS) with 0.25% Tween-20 (TTBS) then probed overnight with an anti-IL-2 antibody. After washing with TTBS, bound antibody was detected with a horseradish peroxidase conjugated anti-rabbit secondary antibody (Abcam) in TTBS. The blots were developed using enzyme chemiluminescence (Pierce Biotechnology, Rockford, IL) and blue X-ray film (Phenix, Hayward, CA).

Western blot analysis of aortic tissues was performed by first mincing the tissue into pieces, rinsing the pieces in PBS, and then homogenizing the minced tissue in ice-cold 4 M urea buffer (100 µl buffer/µg tissue) containing 0.05% Triton X-100 v/v and 1 mM PMSF. The homogenate was sonicated on ice followed by centrifugation to remove insoluble material. The resulting supernatant (extract) was collected, and the protein concentration measured by DC protein assay (Bio-Rad). A 25 µg aliquot was then mixed with an equal volume of 2X Laemmli running buffer containing 5% v/v β-mercaptoethanol. The samples were then analyzed by Western blot as described. Blots analyzing tissues from mice injected with dye-labeled IL-2 were visualized using an Odyssey infrared imager (LI-COR Biosciences).

### Heparinase digestion of tissues

Heparinase I digestion of tissues (spleen, aorta) from Balb/c mice injected with dye-labeled IL-2 was performed by incubating approximately 6, 2×2 mm, pieces of aorta or spleen in digestion buffer (0.03 M Tris-HCl, 3.3 mM calcium acetate, 6 mM sodium acetate, pH 7.0] containing 1 mM PMSF) to which 2 U heparinase I was added and incubated at 37°C for approximately 18 h. The vessel tissue was pelleted by centrifugation and the resultant supernatant collected and total protein present determined by DC protein assay (Bio-Rad). A 50 µg aliquot was mixed with an equal volume of 2X Laemmli running buffer (Bio-Rad) containing 5% v/v β-mercaptoethanol, separated by SDS-PAGE, transferred to nitrocellulose, then visualized on an Odyssey infrared imager (LI-COR Biosciences) or probed with anti-IL-2 antibodies.

### MALDI Analysis

To facilitate MALDI analysis of the 30 kD dimer isolated from EL4-conditioned media, samples of the dimer (25–40 µg) purified by elution from preparative SDS-PAGE (see above) were deglycosylated by treatment with N-glycanase (PNGase-F), O-glycanase, and sialidase as per the manufacture's instructions (GlycoPro Enzymatic Deglycosylation Kit, Prozyme, Hayward, CA). The deglycosylated samples were then subjected to iso-electric focusing on ampholine strips with a pH gradient of 5–8 (Bio-Rad). Multiple focusing strips were run concomitantly followed again by SDS-PAGE permitting some gels to be stained with Coomassie R-250 while others were processed for Western blot analysis with an anti-IL-2 antibody (see above). Using this methodology, focused spots with IL-2 immunoreactivity were identified on the Coomassie-stained gels and excised. The gel pieces were dehydrated by immersion in NaHCO_3_:acetonitrile (1∶1) containing 10 mM DTT, and 55 mM iodoacetamide. The reduced-alkylated samples were re-hydrated with NaHCO_3_ buffer and digested overnight at 37°C with 300 ng of sequencing grade porcine trypsin (Promega). The next day, the supernatants were removed and saved and the gel slices extracted 3X with 50% acetonitrile containing 0.3% TFA v/v. The supernatants were combined, lyophilized to a volume of ∼10 µL, and concentrated using C18 ZipTips (Millipore, Billerica, MA), that were eluted with 95% acetonitrile containing 0.3% TFA v/v. The eluates were then mixed with CHCA matrix (Thermo) and spotted onto a brushed-steel MALDI target plate. Samples were analyzed using a Bruker Autoflex III MALDI-TOF/TOF MS incorporating Bruker LIFT and collision-induced dissociation (CID) fragmentation to generate the protein/peptide sequence for peaks of interest. All data was analyzed by searching the MASCOT server at the Wright State University Proteome Analysis Laboratory.

### Statistics

Software used for statistical analyses was the R library “multicomp”. Specific analyses performed are noted in the figure legends.

## Results

### Dimeric form of IL-2 is present in blood vessels

**Figure 1:**
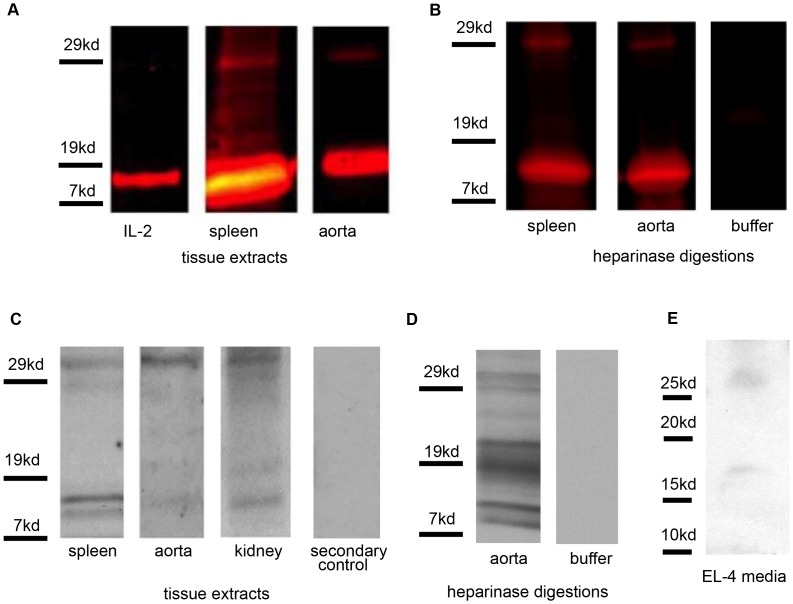
A 30 kD form of IL-2 is present in murine aortas and released by heparinase digestion. (A) Balb/c mice were given 1 µg infrared-IL-2 daily by intraperitoneal injection for 8 doses, and sacrificed 3 days following the last dose. Tissues were processed as described for Western blot analysis, and analyzed on an Odyssey infrared scanner. Results shown are representative of 10 experiments. “IL-2” denotes dye-labeled IL-2 prior to injection. (B) Balb/c mice were given a single dose of 1.5 µg infrared-IL-2, and sacrificed 2.5 days later. Five 1 mm long pieces of murine aortas were incubated at 37°C with 2 U heparinase I or heparinase buffer for 18 h, and the released material was separated by SDS-PAGE and analyzed on an Odyssey infrared scanner. Results shown are representative of 2 experiments. IL-2 was labeled with infrared dye as described in Materials and Methods. (C) Murine spleens, aortas, or kidneys were homogenized, processed for Western blot analysis, and probed with anti-IL-2 antibodies. Results shown are representative of 5 experiments. (D) Murine aortas were digested with heparinase I or buffer alone as above, processed for Western blot analysis, and probed with anti-IL-2 antibodies. Note that dimeric IL-2 appears as a doublet. Results shown are representative of 5 experiments. (E) EL-4 cell supernatants were processed for Western blot analysis and probed with anti-IL-2 antibodies. Results shown are representative of 10 experiments.

Recent work from our laboratory demonstrated that IL-2 is present in blood vessels and is retained there by association with heparan sulfate (HS) [8], [9]. This observation was confirmed, in part, by the systemic administration of infrared-dye labeled IL-2 to Balb/c mice and subsequent recovery of this IL-2 from aortic tissues. The infrared monomeric IL-2, with a MW of approximately 15 kD, was present in aortic tissue homogenates and was released by incubation of aortic tissues with bacterial heparinase I. Interestingly, a 30 kD labeled band was also present in the homogenates and was released by heparinase digestion (Figures 1A, B). Western blot analysis of murine aortic tissues also revealed bands, recognized by anti-IL-2 antibodies, at 15 and 30 kD. These bands were present in both homogenates and in supernatants from heparinase-treated tissue (Figures 1C, D). Sampling of select tissues, including spleen and kidney, revealed the presence of monomeric and dimeric IL-2 in homogenates (Figure 1C and not shown).

We then asked whether a simplified, *in vitro* system such as IL-2 producing cells in culture would generate dimeric IL-2. To this end, EL4.IL-2 cells, a subline producing large amounts of IL-2, were stimulated and supernatants assessed for dimeric IL-2. Both monomeric and dimeric IL-2 were present in supernatants of activated EL-4 cells (Figure 1E). In addition, transglutaminase, noted by Eitan, et al to be important for generating dimeric IL-2, was present in EL4 supernatants by Western blot analysis (not shown). In turn, the γ-glutamyl-ε-lysine epitope, characteristic of transglutaminase-mediated cross-linking, was present in dimeric IL-2 isolated from EL4 supernatants (not shown).

These results suggested that a dimeric form of IL-2 was present in murine tissues. A review of the literature revealed that a dimeric form of IL-2 had previously been identified in the conditioned media of injured fish optic neurons [5], [6], [7] and that this dimeric form of IL-2 induced apoptosis in oligodendrocytes. Given this information, we sought to confirm that this 30 kD band was a dimeric form of IL-2.

### Isolation of dimeric IL-2

Our first step in confirming the presence of a dimeric form of IL-2 was to develop a method for its isolation. Murine aortic tissues or conditioned media from stimulated EL4.IL-2 cells was used as a source of dimeric IL-2. Murine aortic tissues were homogenized in urea, clarified by centrifugation, and the resulting supernatants separated by SDS-PAGE. Supernatants from media of the IL-2 producing lymphoma cells were concentrated and then similarly separated. Companion gels were run for Western blot analysis and zinc-staining. Bands of 15 kD and 30 kD, recognized by a chicken anti-IL-2 polyclonal antibody (Sigma), were excised from the companion zinc-stained gel and electro-eluted. Eluted samples were dialyzed, and the presence of dimeric IL-2 verified by Western blot. Samples of each eluted preparation separated by SDS-PAGE and stained with Coomassie R250 showed the presence of one band per sample.

**Figure 2:**
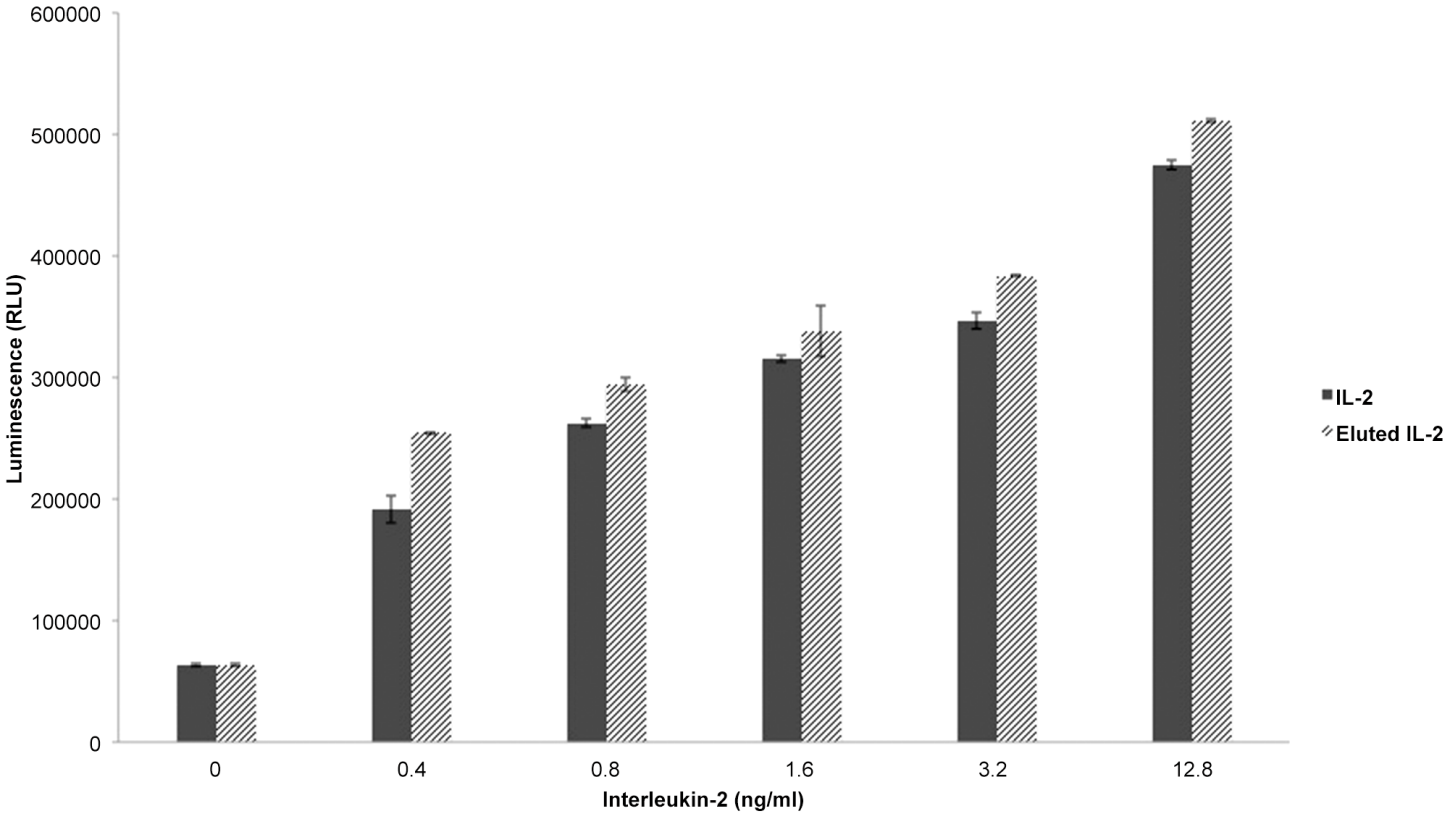
Isolation process does not impact function of IL-2. Recombinant murine IL-2 was isolated in a fashion identical to that used for dimeric IL-2 (eluted IL-2). The proliferative response of CTLL-2 cells to “eluted IL-2” vs IL-2 (taken directly from the vial) was then compared by measuring total ATP content. Results shown are the means ± SD of triplicate wells, and are representative of 3 experiments.

To ensure that the isolation process was not influencing activity, commercial IL-2 was subjected to the same isolation process as described above. As seen in Figure 2, commercial IL-2 and “eluted” commercial IL-2 yielded identical proliferative responses when assayed using an IL-2 dependent cell line, suggesting that the isolation process had no effect on biological activity.

### MALDI analysis of the 30 kD band from EL4 conditioned media confirms the isolation of dimeric IL-2

**Figure 3:**
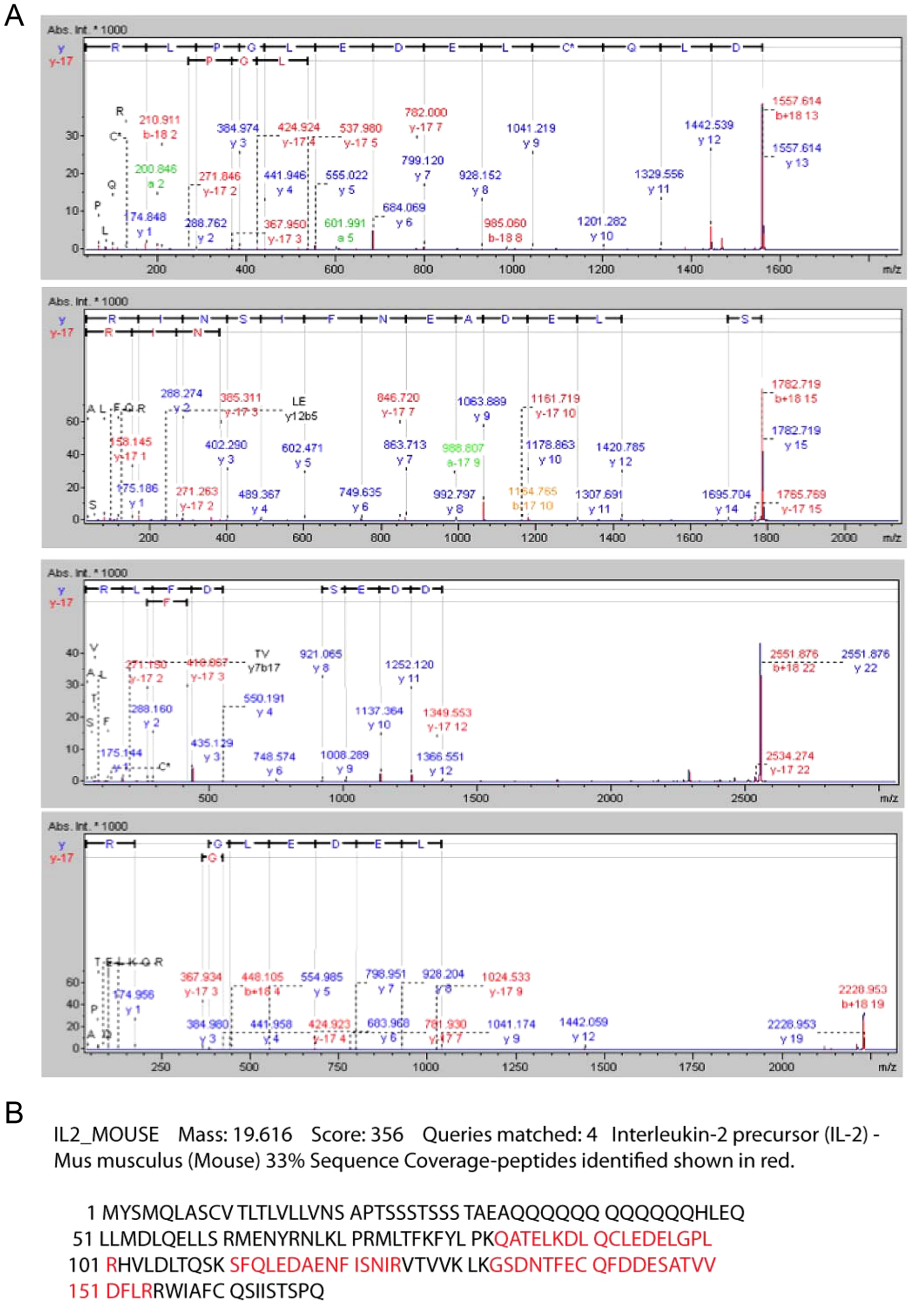
MALDI MS/MS spectra of 4 peaks derived from the tryptic digest of the 30 kD band isolated from EL-4 conditioned media. MS/MS analysis of 30 kD dimer isolated from EL4-conditioned media identified four peaks consisting of 15 to 24 amino acids that yielded a combined score of 356 confirming they came from murine IL-2 (p 0.05).

Our efforts at MALDI analysis of the 30 kD dimer focused on supernatants of murine lymphoma (EL4) cells since these supernatants were less complex than tissue extracts and could be generated in copious amounts. Following fragmentation of the 30 kD dimer (isolated per Methods), MS/MS analysis identified four peaks consisting of 15 to 24 amino acids that were identified as belonging to murine IL-2 (p≤0.05) (Table 1). These peaks yielded a combined score of 356 to confirm the identity as murine IL-2 (Table 1; Figure 3A and B). Sequence coverage of 33.1% was confined to the C-terminal portion of the protein with overlap of 2 of the peptides at a site between 82 and 101 amino acids (Figure 3B).

**Table 1:** MALDI-TOF/TOF analysis of the 30 kD band produced by EL-4 T lymphoma cells and recognized by an anti-IL-2 antibody.

Observed	Expected	Theoretical	Score	Peptide
1557.6212	1556.6139	1556.7504	115	K.DLQCLEDELGPLR.H
1782.7065	1781.6993	1781.8584	123	K.SFQLEDAENFISNIR.V
2227.9432	2226.9360	2227.1154	49	K.QATELKDLQCLEDELGPLR.H
2551.8858	2550.8786	2551.0809	70	K.GSDNTFECQFDDESATVVDFLR.R

IL2_MOUSE Mass: 19616 Score: 356 Queries matched: 4.

Sequence Coverage: 33.1%, pI 4.7.

### Dimeric IL-2 is cytotoxic to multiple cell types

**Figure 4:**
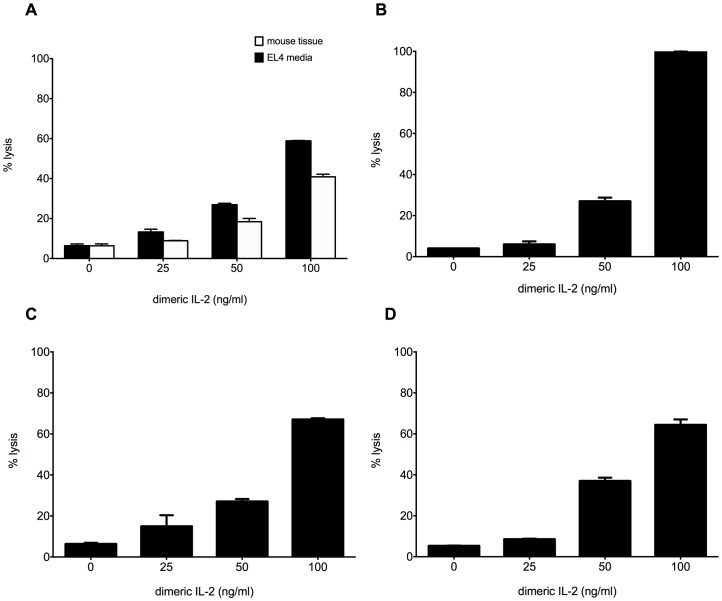
Dimeric IL-2 is cytotoxic to multiple cell types. Dimeric IL-2, isolated from murine aortas or conditioned media from EL-4 T lymphoma cells were added to cultures of (A) primary human vascular smooth muscle cells, (B) normal rat kidney cells (NRK cell line), (C) human renal epithelial cells (HK-2 cell line), (D) murine melanoma cells (B16-F10). The cells were incubated for 2 h with increasing concentrations of dimeric IL-2. Cytotoxicity was assessed by release of LDH and expressed as % lysis (experimental/total lysis x 100). Smooth muscle cells cultured under identical conditions with either 1000 ng/ml eluted, commercial, murine IL-2 or eluate from an empty gel slice each yielded a % lysis of 2.9%, and 3.2% respectively. Results shown are the means ± SD of triplicate wells, and are representative of 5–20 experiments. Percent lysis is significantly different across the 4 concentrations of dimeric IL-2 in each figure (A–D, p = 4.8×10^−7^, p = 3.2×10^−7^, p = 1×10^−4^, p = 1.8×10^−10^, by ANOVA).

We next sought to determine whether the function of dimeric IL-2 was similar to or different than the monomeric form. Given the work by Eitan, et al, we focused on the potential cytotoxicity of dimeric IL-2. Since we identified dimeric IL-2 in arterial tissues, and previously demonstrated that VSMC express IL-2Rβ [8] we asked whether dimeric IL-2 was cytotoxic to VSMC. To this end, dimeric IL-2 was isolated from murine aortas or EL-4 culture media as described above. Increasing concentrations of dimeric IL-2 were added to cultures of VSMC isolated from human aortas. Cytotoxicity was assessed by release of lactate dehydrogenase (LDH). As seen in Figure 4A, dimeric IL-2 induced rapid cell death of VSMC in a dose-dependent fashion. Given this response, we asked if dimeric IL-2 was cytotoxic to other cell types. Dimeric IL-2 was cytotoxic to several cell types expressing the IL-2R, including human and rat kidney epithelial cells [12], [13], human and murine melanoma cells [14], [15], murine lymphocytes, murine lymphoma cells, human leukemia cells, and human colon carcinoma cells [16] (Figure 4B–D and not shown). Of note, neither eluted monomeric IL-2 up to 1000 ng/ml, nor the eluate from an empty gel slice, were cytotoxic (see Figure 4 legend).

Given these results, we asked whether recombinant, murine IL-2 could be used to generate a cytotoxic dimer. Using transglutaminase, commercially available monomeric IL-2 was provided as a substrate to generate cross-linked 30 kD dimers, which were then isolated as previously described. Dimeric IL-2 originating from bacterial sources, and therefore not glycosylated, was not cytotoxic. Similarly, dimeric IL-2 originating from insect sources (less complex than mammalian glycosylation [17]) also lacked cytotoxicity. Together, these results suggest that glycosylation contributes to the cytotoxicity of dimeric IL-2.

**Figure 5:**
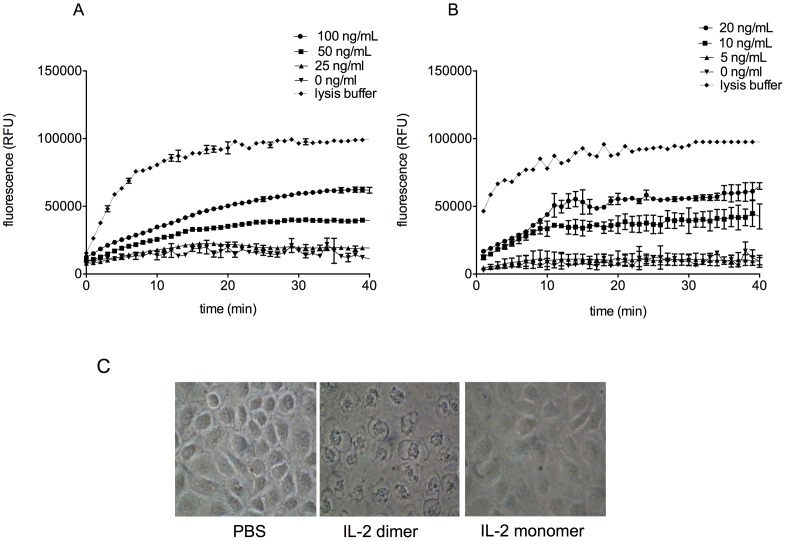
Dimeric IL-2 induces rapid onset of cell death. (A, B) CellTox Green dye was added to cultured VSMC followed by increasing concentrations of dimeric IL-2 isolated from murine aortas (A) or EL4 media (B). Tissue culture plates were immediately placed in the microplate reader and excited at 485 nm per manufacturer's instructions. Cells were lysed, as a positive control, using a proprietary lysis buffer (Roche). In part A, 100 ng/ml and 50 ng/ml are significantly different than 0 ng/ml (p = 1×10^−4^ for each, by Dunnett's test for multiple comparisons). In part B, 20 ng/ml and 10 ng/ml are significantly different than 0 ng/ml (p = 1×10^−4^ for each, by the same analysis). (C) Normal rat tubular epithelial cells (NRK-52E) were cultured for 30 minutes with 20 ng/ml dimeric IL-2, 40 ng/ml monomeric IL-2, or PBS. Scale bar: 50 µm. Results are representative of 10 experiments.

The results of the LDH release assay demonstrated that dimeric IL-2 kills cells rapidly. To obtain a more precise measure of this effect, especially at early time points, we evaluated cell death using a fluorescent reagent that binds DNA and is designed to assess cell death kinetically. As seen in Figures 5A and B, the onset of cell death was very rapid; half of the maximal fluorescence induced by dimer-mediated cytotoxicity was reached approximately 10 minutes after exposing VSMCs to dimeric IL-2.

The rapidity of dimer-induced cell death suggested that the mechanism might involve oncosis rather than apoptosis, since apoptosis typically occurs over hours rather than minutes. Phase contrast microscopy of kidney epithelial cells exposed to dimeric IL-2 for 30 minutes revealed membrane blebbing consistent with an oncotic form of cell death (Figure 5C).

### Inhibition of dimer-mediated cytotoxicity

**Figure 6:**
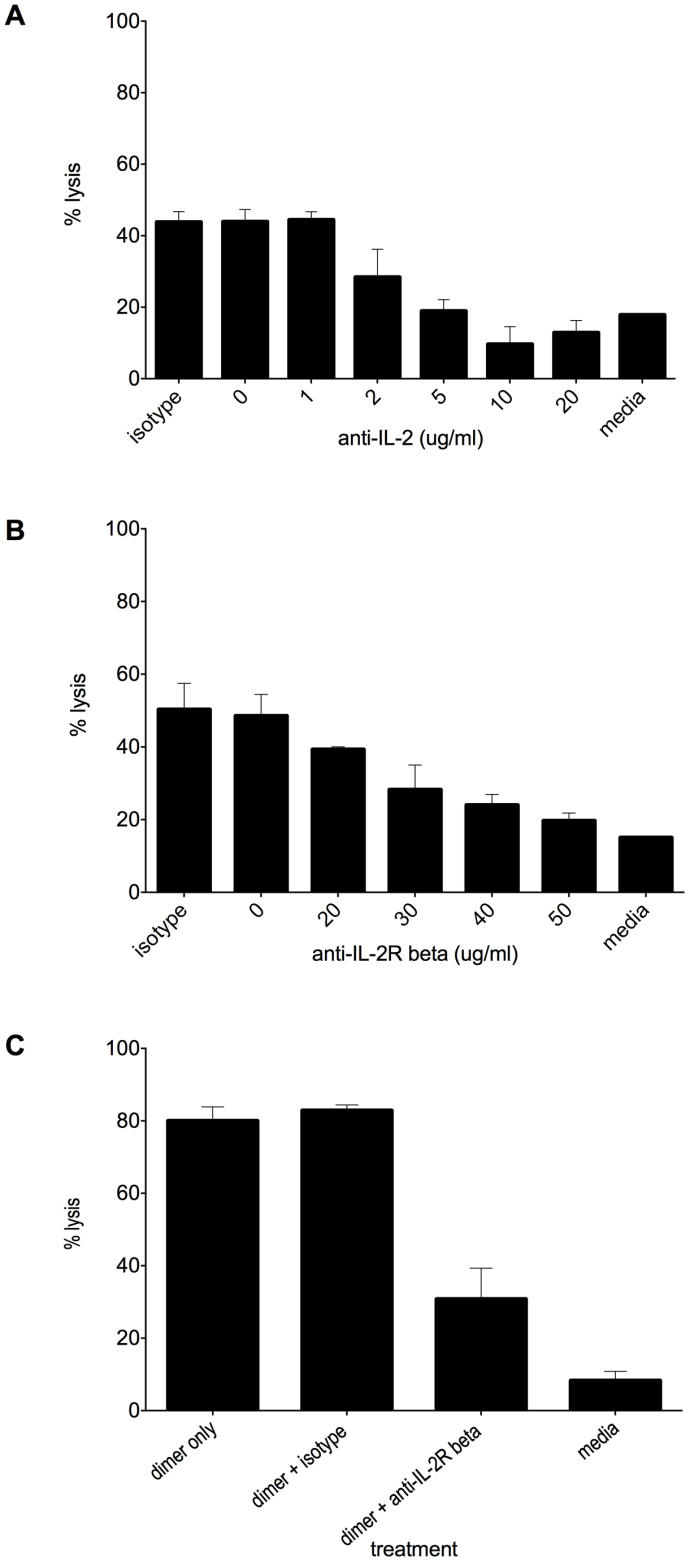
Dimeric IL-2-mediated cell death is blocked by antibodies recognizing IL-2 or IL-2Rβ. (A) Murine dimeric IL-2, from tissues, at 100 ng/ml was incubated with increasing concentrations of anti-murine IL-2 antibodies (clone S4B6) or an isotype control (20 µg/ml) for 30 minutes at 37°C then added to cultured VSMC. Cell death at 1 hour post-dimer addition was assessed by CellTox Green as previously described. “Media” indicates cells cultured in the absence of dimer or antibody. (B) VSMCs were pre-incubated for 15 minutes with increasing concentrations of anti- murine IL-2Rβ antibodies or an isotype control (50 µg/ml). Dimeric IL-2 at 100 ng/ml was added and cell death assessed using CellTox Green. Cell death at 20 minutes post-dimer addition is shown. (C) Rat tubular epithelial cells were pre-incubated for 15 minutes with 10 µg/ml anti-rat IL-2Rβ antibodies or an isotype control (10 µg/ml). Dimeric IL-2, from EL-4 media, at 10 ng/ml was added and cell death was assessed at 20 minutes post-dimer addition as in B. The results shown are the mean ± SD of duplicate wells, and are each representative of 5 separate experiments. Percent lysis is significantly different across the increasing concentrations of antibodies in Figures A and B (A, p = 6.6×10^−6^; B, p = 5.8×10^−5^, by ANOVA). In C, treatment with anti-CD122 is significantly different than isotype control (p<.05, one-sided t test).

To begin to address the mechanism of dimer-induced cell death, we first asked whether anti-IL-2 antibodies could inhibit dimer-mediated cytotoxicity. For these studies we chose S4B6, a well-described anti-IL-2 antibody known to neutralize IL-2-mediated proliferation [18]. As seen in Figure 6A, incubation of the dimer with S4B6 blocked dimer-mediated cell cytotoxicity in a dose-dependent fashion. This result suggests that the peptide sequence within IL-2 that mediates proliferation is exposed in the dimeric form and is likely important for dimer-mediated cytotoxicity.

We next asked whether interference with the IL-2 receptor would alter dimer-mediated cell death. Little information exists about the expression of the IL-2 receptor in VSMCs. Previous studies from our laboratory indicate that these cells express at least the β portion of the IL-2 receptor [8]. To assess the role of the IL-2R in dimeric IL-2 mediated cell death, we pre-incubated human VSMCs with a polyclonal anti-IL2Rβ antibody and then exposed them to dimeric IL-2. As seen in Figure 6B, pre-incubation with anti IL-2Rβ antibodies inhibited dimer-mediated cell death in a dose dependent fashion, suggesting that dimer-mediated cell death occurs through association with IL-2Rβ. Similar results were obtained in rat kidney epithelial cells using a monoclonal anti-rat anti-IL2Rβ antibody (Figure 6C) and melanoma cells (not shown). Whether the α and γ portions of the IL-2R are also involved in dimer-mediated cell death is currently under investigation.

## Discussion

This report demonstrates, for the first time, the identification and isolation of a dimeric form of IL-2 from mammalian tissues. The cytotoxicity of dimeric IL-2 stands in stark contrast to the proliferative and survival functions of the monomer. This finding leads to several questions concerning the function and regulation of the dimeric form.

While the proliferative properties of IL-2 have been recognized since its discovery, the importance of IL-2 in cell death was revealed after the generation of IL-2 knockout mice, which develop a lymphoproliferative disorder [4]. At approximately the same time, IL-2 was shown to induce apoptosis when activated T cells were exposed to antigen and IL-2 [2]. Activation-induced cell death, mediated by IL-2, was therefore identified as a mechanism through which IL-2 induces cell death [19]. In light of our findings, we suggest that dimeric IL-2 also contributes to IL-2-mediated cell death *in vivo*.

During an immune response, activated T cells produce both glycosylated and non-glycosylated IL-2. *In vitro* studies have shown that the ratio of glycosylated to non-glycosylated IL-2 produced by human peripheral blood lymphocytes depends on the stimulus and duration of stimulation [20]. In the study by Conradt, et al, human peripheral blood lymphocytes stimulated with A23187 and PMA initially produced non-glycosylated IL-2, and only produced glycosylated IL-2 30–40 h after stimulation. Glycosylation of IL-2 appears to be important since both unglycosylated (bacterial) and simply glycosylated (insect) forms of dimeric IL-2, generated from recombinant sources, are not cytotoxic. Whether glycosylation contributes to conformation of the dimer, binding to the receptor, or other, is not known. The role of glycosylation in dimer-mediated cell death is currently under investigation.

The presence or absence of glycosylation likely represents one means by which the cytotoxicity of dimeric IL-2 is controlled. The aforementioned *in vitro* data suggests one possible scenario. *In vivo*, T cells activated by foreign antigen initially produce non-glycosylated IL-2. As the immune response proceeds, glycosylated IL-2 is produced, resulting in the generation of cytotoxic, dimeric IL-2 that kills IL-2R+ cells and restricts their excess accumulation. Alternatively, the later production of glycosylated IL-2 may serve to replenish stores released by heparanase earlier in the immune response.

The potency of dimeric IL-2 suggests that it is tightly regulated *in vivo*. In addition to glycosylation, other mechanisms to control dimeric IL-2 include IL-2R expression, heparanase expression, and the presence/absence of transglutaminase. Our results indicate that antibodies recognizing IL-2Rβ block the cytotoxicity of dimeric IL-2. Interestingly, IL-2Rβ also dimerizes, and this property may facilitate association with dimerized IL-2 [21]. Studies addressing whether IL-2Rα or γ are involved in the cellular response to dimeric IL-2 are in progress.

Although produced by most mammalian cells, heparanase is tightly controlled by several mechanisms, including (1) regulated secretion, (2) uptake of secreted heparanase by low-density lipoprotein-related receptor protein [22] and syndecan-4 [23], and (3) expression, in that only one functional isoform of mammalian heparanase has been identified [16]. Since dimeric IL-2 is released by heparanase, tight control of heparanase expression would, in turn, regulate the release of dimeric IL-2. Whether other matrix-degrading enzymes, such as matrix-metalloproteinases, release dimeric IL-2 is currently under investigation.

Studies by our laboratory and Eitan, et al, indicate that cytotoxic, dimeric IL-2 is generated by transglutaminase-mediated crosslinking of monomeric IL-2. Transglutaminases are ubiquitous, well-described cross-linking enzymes known to alter the functions of proteins through oligomerization. Probably the best-studied example is the crosslinking of fibrinogen or fibrin [24] in the formation of blood clots. Other examples include the dimerization of osteopontin, which increases its adhesivity to osteoclasts as compared to the monomer [25], and the oligomerization of sonic hedgehog, which increases its bioactivity as a morphogen [26]. The functional differences conferred by dimerization of IL-2, however, may represent one of the most distinct, transglutaminase-mediated changes in a biologically active protein.

Our finding that dimeric IL-2 is cytotoxic to several parenchymal cell types begs the question as to its function *in vivo* with respect to these cells. Given that dimeric IL-2 is released by heparanase and cross-linked by enzymes such as transglutaminase, its presence *in vivo* may be limited to or concentrated in areas of injury or inflammation. Because dimeric IL-2 appears to precipitate a necrotic vs apoptotic cell death, we anticipate that dimeric IL-2 would serve to propagate inflammation.

In summary, our laboratory has identified a dimeric form of IL-2 present in mammalian tissues. This form of IL-2 causes a very rapid cell death, which appears to be oncotic rather than apoptotic. These findings yield several questions regarding the mechanism of cell death mediated by dimeric IL-2, its regulation, and the contribution of dimeric IL-2 to cellular homeostasis in both immune and non-immune systems. These answers, as they unfold, may dramatically alter our perception of how IL-2 functions *in vivo*.
